# Comparison of a microsphere-based platform with a multiplex flow cytometric assay for determination of circulating cytokines in the mouse

**DOI:** 10.1186/s12907-017-0068-6

**Published:** 2018-01-06

**Authors:** Alain Stricker-Krongrad, Catherine Shoemake, Miao Zhong, Jason Liu, Guy Bouchard

**Affiliations:** Sinclair Research Center, LLC, 562 State Rd. DD, Auxvasse, MO 65231 USA

## Abstract

**Background:**

Measuring expression profiles of inflammatory biomarkers is important in monitoring the polarization of immune responses; therefore, results should be independent of quantitation methods if they are to be accepted as validated clinical pathology biomarkers. To evaluate effects of differing quantitation methods, the seven major circulating Th1/Th2/Th17 cytokines interleukin 2 (IL-2), interferon γ (IFN-γ), tumor necrosis factor α (TNF-α), IL-4, IL-6, IL-10 and IL-17A were quantified in plasma of lipopolysaccharide (LPS)-treated mice with two different multiplex platforms.

**Methods:**

Female C57BL6 mice were treated orally with vehicle or dexamethasone, followed by LPS intravenously. Plasma samples were analyzed 0.5, 1, 2, 4, and 6 h post-LPS challenge with assays at Myriad-RBM and compared to assays performed on a BD Accuri C6 flow cytometer.

**Results:**

IL-17A response to LPS was limited but sustained, and the response for the remaining cytokines were early and transient; dexamethasone reduced expression of all 7 cytokines. TNF-α and IL-6 levels were similar across both assays, and IL-4 levels were generally very low. Plasma levels of remaining cytokines were variably lower with BD assays than Myriad-RBM assays.

**Conclusions:**

The present findings demonstrate that quantitation of circulating biomarkers of inflammation can be achieved using multiplexed flow cytometry, but careful consideration must be taken for assay validation when cross-referencing with another multiplexed assay.

## Background

A number of cytokines, such as interleukin 2 (IL-2), interferon γ (IFN-γ), tumor necrosis factor α (TNFα), IL-4, IL-6, IL-17A and IL-10, become elevated in tissues in response to inflammation. These cytokines are also key regulators of immune responses and elevations of these different cytokines are individually associated with responses of specific T helper cell (Th) lineages. IFN-γ, IL-2, and TNFα are associated with the Th1 response. IL-4, IL-6 and IL-10 are associated with the Th2 response. IL-6 and IL-17A are associated with the Th17 response. Since these variabilities exist, measuring expression profiles of these cytokines is important to monitor the polarization of the immune response.

Lipopolysaccharide (LPS) administration reliably induces an acute inflammation that is associated with increases of a number of inflammatory cytokines in the peripheral blood of LPS-treated animals [1]. Additionally, dexamethasone (DEX) administration inhibits the effects of LPS on cytokine synthesis in animal models [2]. These properties of LPS and DEX can then be used for evaluation of anti-inflammatory drug effects or, as in the present case, comparison and validation of cytokine identification and quantitation methods. The objective of the present study was to implement and validate bench top flow cytometry for the ex vivo quantitation of circulating cytokine levels in multiplex assay formats. Cytokine quantitation was performed on a Becton Dickson (BD) Accuri C6 flow cytometer, and results were compared to those produced by a commercial biomarker testing laboratory performing rodent multi-analyte profile (MAP) assays.

## Methods

### Test system

Female C57BL/6 mice, approximately 6–9 weeks of age and weighing 17–20 g, were purchased from Charles River Laboratories. The mice were housed 2–3 per cage in shoebox cages in a room with temperature maintained between 64 and 80 °F (18-29 °C) and with a 12-h light/12-h dark photoperiod. The animals had ad libitum access to Harlan Teklad Global Rodent Diet 2018 and deep well water. All study procedures were reviewed and approved by Sinclair Research Center’s Institutional Animal Care and Use Committee. Housing and animal care conformed to the guidelines of the Guide for the Care and Use of Laboratory Animals, 8th edition published by the U.S. National Institutes of Health and to applicable institutional standard operating procedures. Euthanasias were performed in accordance with the American Veterinary Medical Association’s published guidelines [3].

After being acclimated for 3 days, mice were randomized into groups with 6 mice in the untreated group, 32 mice in a group treated with LPS, and 32 mice in a group treated with LPS plus DEX. Identification of each animal was maintained using ear notches and cage cards.

Methyl cellulose, DEX, and LPS were obtained from Sigma-Aldrich (St Louis, Missouri). Methyl cellulose was dissolved in sterile water (Hospira, Lake Forest, Illinois) overnight to form a 0.5% solution for use as the vehicle. DEX was suspended overnight in 0.5% methyl cellulose at a concentration of 0.5 mg/mL and then sonicated briefly before dosing. LPS was prepared the day before dosing in 0.9% saline for injection (Hospira) at a concentration of 0.04 mg/mL.

Mice in the untreated group were bled for plasma without any treatment. Mice in the LPS treatment group were administered 0.5% methyl cellulose at 10 mL/kg via oral gavage, and then were treated 1.5 h later with 0.2 mg/kg LPS intravenously (IV) at a volume of 5 mL/kg. The LPS plus DEX groups were administered 5 mg/kg dexamethasone via oral gavage, and then underwent the same IV LPS treatment as above 1.5 h later. Six to eight mice from each treatment group were bled for plasma at 0.5, 1, 2, 4, and 6 h following LPS challenge.

### Plasma preparation and analysis

All blood samples were collected into K_2_EDTA tubes (0.5 mL, Greiner Bio-One North America, Inc. Monroe, North Carolina). Filled tubes were placed on wet ice and were processed within 30 min after blood collections. The samples were centrifuged at 3000 rpm for 15 min at 4 °C; plasma was then drawn off and placed into separate vials. Plasma samples were separated into two sets and placed on dry ice and stored at −70 °C before being analyzed for cytokine profiles.

One set of plasma samples were shipped on dry ice to Myriad RBM, Inc. (Austin, TX) for cytokine profiling with Mouse Cytokine Panels A & B (4-h time point) and Rodent MAP V3.0 Antigen (0.5-, 1, 2, and 6-h time points) assays (based on a Multiplexed Luminex Platform).

Seven cytokines (IL-2, IFN-γ, TNFα, IL-4, IL-6, IL-17A, and IL-10) were analyzed at the 2- and 4- h time points in the second set of collected plasma samples with a cytometric bead array (CBA) mouse Th1/Th2/Th17 cytokine kit (BD Biosciences) on a BD Accuri C6 flow cytometer. The CBA Mouse Th1/Th2/Th17 Cytokine Kit Manual (BD Biosciences) was followed for the assay procedure. Plasma samples were thawed at room temperature and then placed on wet ice for duration of analysis. One vial of mixed standards was freshly reconstituted in 2.0 mL of assay diluent, and then was serial diluted. The concentrations of standards for each cytokine were 0, 20, 40, 80, 156, 312.5, 625, 1250, 2500, and 5000 pg/mL. Seven types of cytokine capture beads were freshly mixed in equal amounts (10 μL bead per assay tube) in a master tube. To perform the assay, 50 μL of the mixed beads were incubated with 50 μL of standards or samples along with 50 μL of Phycoerythrin (PE) Detection Reagent in a MultiScreen filter plate (1.2 μm pore size, EMD Millipore, Darmstadt, Germany) at room temperature for 2 h. At the end of incubation, the plate was drained on a vacuum manifold. The beads in each of the individual wells of the plate were resuspended in 120 μL of wash buffer, and were then analyzed on the BD Accuri C6 flow cytometer. The seven distinct fluorescence beads were sorted with fluorescence signals captured in FL4 channel. PE intensity of individual beads was captured in FL2 channel. Approximately 200 events for each bead group were acquired (based on experience in generating data in previous experiments). The acquired data were subsequently analyzed for individual cytokine concentrations in each sample using the FCAP Array software (BD Biosciences).

Results for the 7 cytokines IL-2, IFN-γ, TNFα, IL-4, IL-6, IL-17A, and IL-10 were compared between BD Biosciences and Myriad RBM assays. Concentrations for individual cytokines were expressed as mean ± standard deviation. Effects of DEX on LPS-induced plasma cytokine changes were evaluated with a two-way student t-test.

## Results

### Levels of the selected cytokines in plasma of normal mice

For flow cytometry using BD CBA, a set of mixed standards for the 7 cytokines (IL-2, IFN-γ, TNF-α, IL-4, IL-6, IL-17A and IL-10) were freshly prepared with serial dilution. When recombinant standards were diluted in normal mouse plasma, the data showed that each of the 7 cytokines was quantitated in the linear range between the expected concentrations of 20–5000 pg/mL. Circulating concentrations of the same cytokines were measured in 6 untreated normal mouse plasma samples. None of the 7 cytokines could be detected or quantified with the CBA or the Myriad RBM assays in the normal plasma, indicating that the background of cytokine levels were below the lower limit of detection.

### Time-course of cytokines stimulation after LPS administration

The pharmacodynamics effects of LPS on Th1/Th2/Th17 circulating cytokines as quantitated with the Myriad RBM Assay are indicated in Table 1. After acute IV administration of 0.2 mg/kg LPS in mice, classical stimulatory responses were observed with a TNF-α peak at 1–2 h, followed by peaks of IFN-γ, IL-10 and IL-6 at 2 h, and gradual decline over the following 4 h. [4]. These time course data were used to select two critical time points, 2- and 4-h post LPS stimulation, to conduct further comparative analyses of the two analytical methods.

**Table 1:** Time Course Effects of LPS on Th1/Th2/Th17 Cytokine Plasma Levels in LPS-Treated Mice Quantitated with Myriad Assay

	Time post-LPS exposure				
Cytokines	0.5^a^ Hours	1.0^a^ Hours	2.0^a^ Hours	4.0^b^ Hours	6.0^a^ Hours
IL-2 (pg/mL)	–	–	245	68	–
IL-4 (pg/mL)	–	–	–	80	–
IL-6 (pg/mL)	176	4597	24,667	2936	1066
IL-10 (pg/mL)	697	5013	7717	3878	1646
IL-17A (ng/mL)	–	0.025	0.083	0.080	0.031
INF-γ (pg/mL)	–	447	1347	287	341
TNF-α (ng/mL)	0.22	1.82	0.42	0.40	0.15

^a^n = 6, ^b^n = 8

### Comparative levels of the selected cytokines in plasma of LPS treated mice

For flow cytometry using BD CBA, the 7 cytokines were detected in diluted plasma for the 2-h samples and in undiluted plasma for the 4-h samples. IL-2 and IL-4 were below the lower limit of detection (LLOD) in both 2- and 4- h samples (data not shown). IL-17A was detectable only in the 4-h samples, but was below the lower limit of quantification (LLOQ). The other 4 cytokines (IFN-γ, TNF, IL-6, and IL-10) were detected within the defined concentration ranges. IL-6 was the only cytokine that needed to be quantitated in the diluted plasma. It was shown that 10× dilution was appropriate for the 2-h samples and 5× dilution potentially for the 4-h samples. Variations in concentrations of cytokines were consistent and acceptable when determined in diluted plasma samples with BD CBA assay. Minimum required dilution was evaluated at the 2-h time point since the linear range of the BD CBA assay was limited to 20–5000 pg/mL. Coefficient of variations for dilutions of IL-10, TNF-α, and IL-6 were 12.5%, 11.2%, and 11.1% respectively in the LPS treated samples, and were 2.5%, 5.5%, and 6.2% respectively in the LPS plus DEX treated samples (Table 2).

**Table 2:** Quantitation of Circulating IL-6, IL-10, & TNF-α in Diluted 2-Hour Plasma Samples with the BD CBA Assay

	Treatments							
	LPS				LPS + Dexamethasone			
Cytokines	10× dilution	5× dilution	2× dilution	%CV^a^	10× dilution	5× dilution	2× dilution	%CV^a^
IL-6 (pg/mL)	33,926	39,860	32,419	11.1	10,537	11,032	11,906	6.2
IL-10 (pg/mL)	344	426	435	12.5	351	342	334	2.5
TNF-α (ng/mL)	1.8	2.2	2.3	11.2	0.2	0.2	0.3	5.5

^a^%CV = coefficient of variation

In the Myriad RBM platform, while LPS was shown to increase plasma TNF-α to similar levels in both 2- and 4- h samples (Table 3), DEX had much weaker inhibition in 4-h samples than in 2-h samples (Table 4). Plasma IFN-γ was increased as previously reported [1] following LPS challenge in the Myriad RBM assays, while an increase did not occur in the BD CBA assay (Tables 3 and 4). IL-10 was quantified at lower plasma levels with a less potent inhibition of DEX in BD CBA assay than in Myriad-RBM assay.

**Table 3:** Concentrations of the Circulating Th1/Th2/Th17 Cytokines in LPS-Treated Mice Quantitated with Myriad and BD CBA Assays

	Time post-LPS exposure			
	2 h		4 h	
Cytokines	Myriad	BD CBA	Myriad	BD CBA
IL-2 (pg/mL)	245	–	68	–
IL-4 (pg/mL)	–	–	80	–
IL-6 (pg/mL)	24,667	35,402	2935	5925
IL-10 (pg/mL)	7717	402	3878	238
IL-17A (ng/mL)	0.083	–	0.080	–
INF-γ (pg/mL)	1347	0.8	287	41
TNF-α (ng/mL)	0.42	2.09	0.40	0.35

**Table 4:** Concentrations of the Circulating Th1/Th2/Th17 Cytokines in LPS plus Dexamethasone-Treated Mice Quantitated with Myriad and BD CBA Assays

	Time post-LPS exposure			
	2 h		4 h	
Cytokines	Myriad	BD CBA	Myriad	BD CBA
IL-2 (pg/mL)	120	–	68	–
IL-4 (pg/mL)	–	–	53	–
IL-6 (pg/mL)	6482	11,158	314	629
IL-10 (pg/mL)	4060	343	1438	164
IL-17A (ng/mL)	0.037	–	0.020	–
INF-γ (pg/mL)	690	–	82	–
TNF-α (ng/mL)	0.11	0.20	0.18	0.05

**Fig. 1:**
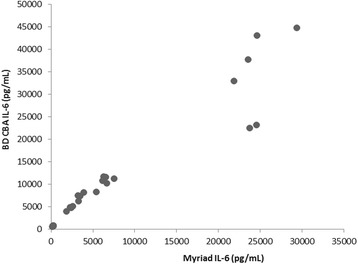
Comparison between Myriad and BD CBA assays in measuring individual circulating IL-6 levels in mice exposed to acute LPS administration with/without dexamethasone suppression

In a comparison of both the Myriad RBM and BD Biosciences multiplex platforms, DEX was shown to inhibit plasma concentrations of IFN-γ, TNF-α, IL-6, and IL-10 in both 2- and 4- h samples. A similar inhibitory effect of DEX was also observed for IL-17A in the 4-h samples. However, variations were observed between the two assay platforms in terms of cytokine concentrations, time course effects of LPS, and magnitudes of DEX inhibition. IL-6 was the only cytokine that was detected comparably with Myriad-RBM assays and BD CBA assay, as demonstrated by the direct relationship between the two assays (Fig. 1).

## Discussion

**Fig. 2:**
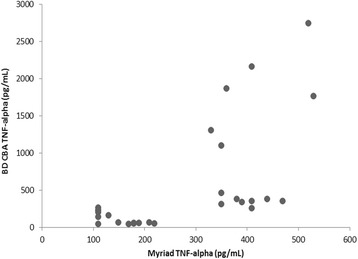
Comparison between Myriad and BD CBA assays in measuring individual circulating TNF-α levels in mice exposed to acute LPS administration with/without dexamethasone suppression

Plasma TNF-α has previously been shown to peak at 1 h post-LPS challenge and then to gradually decrease over time in treated mice [5]. In the BD CBA assay, a similar LPS effect on TNF-α was observed in the 2- and 4- h plasma samples, and the DEX inhibitions were comparable between the 2- and 4- h plasma samples. In the Myriad-RBM platform, while LPS was shown to increase plasma TNF-α to similar levels in both the 2- and 4- h samples, DEX had much weaker inhibition in the 4-h samples than in the 2-h samples. Therefore, this would suggest that the BD CBA assay was more accurate in measuring biologically-relevant TNF levels than Myriad RBM assays. In addition, this is supported by the lack of direct relationship between the two assays as illustrated in Fig. 2.

Plasma IFN-γ was shown to increase through 4 h post-LPS challenge in treated mice [1]. A similar LPS effect on IFN-γ was observed in the 2- and 4- h plasma samples with the BD CBA assay, whereas an opposite trend for the IFN-γ secretion was observed in the same samples with the Myriad RBM assays.

**Fig. 3:**
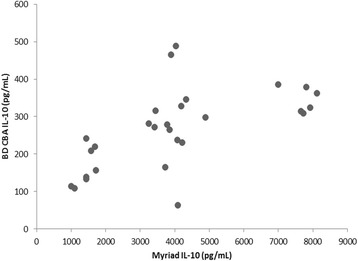
Comparison between Myriad and BD CBA assays in measuring individual circulating IL-10 levels in mice exposed to acute LPS administration with/without dexamethasone suppression

Plasma IL-10 was quantified at lower levels in the BD CBA assay than in Myriad RBM assays. DEX was shown to be less potent to inhibit IL-10 with BD CBA assay than with Myriad RBM assays. No other differences were found for IL-10 quantification between the two assay platforms, although the relationship between the two assays was weak (Fig. 3). LPS and DEX are frequently used in rodent studies evaluating various inflammatory diseases, responses, and chemical or medical agents. Their respective effects and responses in various scenarios have been described in publications such as those by NO Al-Harbi, F Imam, MM Al-Harbi, MA Ansari, KM Zoheir, HM Korashy, MM Sayed-Ahmed, SM Attia, OA Shabanah and SF Ahmad [6].

In the BD CBA assay, the time course effect of LPS on plasma TNF-α was consistent with what was previously reported [5], and the DEX inhibitions were comparable between the 2- and 4- h plasma samples. Reproducible circulating IL-6 was obtained for plasma samples of the LPS treated mice with the assays from both Myriad-RBM and BD Biosciences. IL-6 was the only cytokine that was quantified comparably between the BD CBA and the Myriad-RBM assays, and also the only cytokine that needed be quantitated in diluted plasma when using the BD CBA assay, an indication of a high level of stimulation. IL-4 was the signature Th2 cytokine [7] which was supposed to not be induced by LPS treatment. The lack of signal in IL-4 quantification with BD CBA assay reflected the specificity of this kit in IL-4 measurement.

The BD CBA cytokine assay was not as sensitive as the Myriad RBM assays in detecting and quantitating circulating IL-2, IL-10, and IL-17A levels in the LPS treated mice, but was more biologically-accurate in measuring circulating IL-4, TNF-α, and IFN-γ levels. Differences and similarities between these two assays may relate to the format of these multiplexed assays but also to the nature of the immunological reagents used to capture and detect these cytokines. Although unknown at this time, it is quite possible that the antibodies used for the two assay platforms are identical when IL-6 is considered and different when TNF-α and IL-10 are measured.

There are well-accepted methods for the validation of biomarkers [8, 9], although some form of consensus still needs to be reached on standardization and validation of multi-parametric flow cytometry assays [10] and there are challenges surrounding both clinical specimen analysis and technical variations between instruments [11]. As a general rule, multiplex cytokine assays are cross-validated with or referenced to single analyte immunoassays [12] and more studies comparing different multiplex platform are needed to enable users to determine which are best for a particular study [13]. Previous studies have highlighted the intrinsic differences in reproducibility and accuracy between these technologies [14, 15] and our present report supports the current notion that careful consideration must be taken before generalization of biomarker clinical data when generated on a specific multiplex platform.

## Conclusion

In conclusion, reproducible quantitation of circulating TNF-α and IL-6 levels were obtained from plasma samples of LPS treated mice with assays from both Myriad RBM and BD Biosciences. The BD CBA cytokine assay was not as sensitive as the Myriad RBM assays in detecting and quantitating circulating IL-2 and IL-4 and IL-17A levels in the LPS treated mice, but was more sensitive in measuring circulating IFN-γ levels. Reliable circulating IL-4 measurements were not achieved by either assay. The present data demonstrate that the quantitation of circulating biomarkers of inflammation can be achieved using multiplexed flow cytometry, but that careful considerations have to be made to the biological validation of the assays. This data also suggest that a multiplex assay cannot be used as a validation reference when implementing another multiplex assay on a different platform.

## Abbreviations

BD: Becton Dickson
CBA: Cytometric bead array
DEX: Dexamethasone
IFN-γ: Interferon-γ
IL: Interleukin
IV: Intravenously
LLOD: Lower limit of detection
LLOQ: Lower limit of quantification
LPS: Lipopolysaccharide
MAP: Multi-analyte profile
PE: Phycoerythrin
Th: T helper cell
TNF-α: Tumor necrosis factor-α
